# Supplementary figures and images for: Conversion of alpine pastureland to artificial grassland altered CO2 and N2O emissions by decreasing C and N in different soil aggregates
Source: PeerJ. 2021 Jul 14;9:e11807. doi: 10.7717/peerj.11807 (PMC8759380; doi:10.7717/peerj.11807)

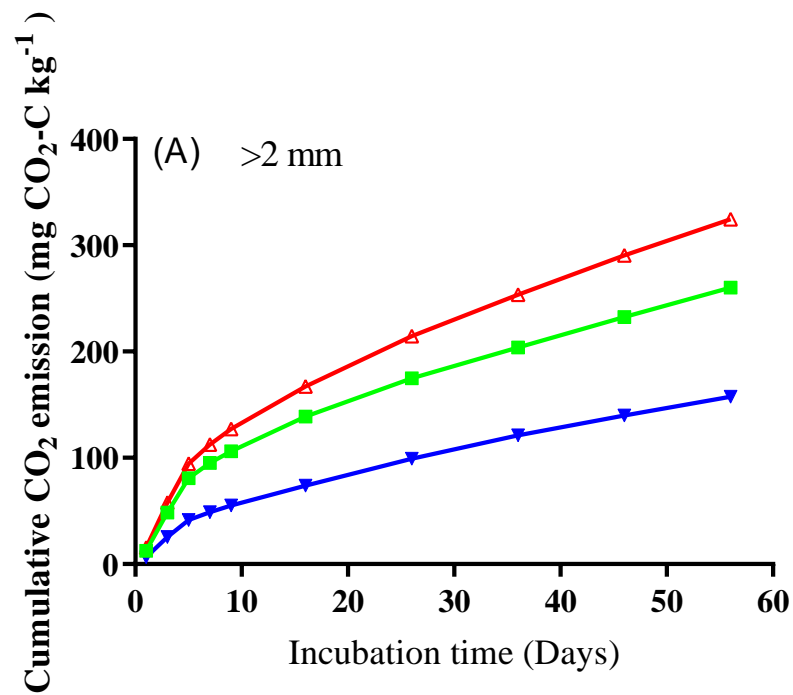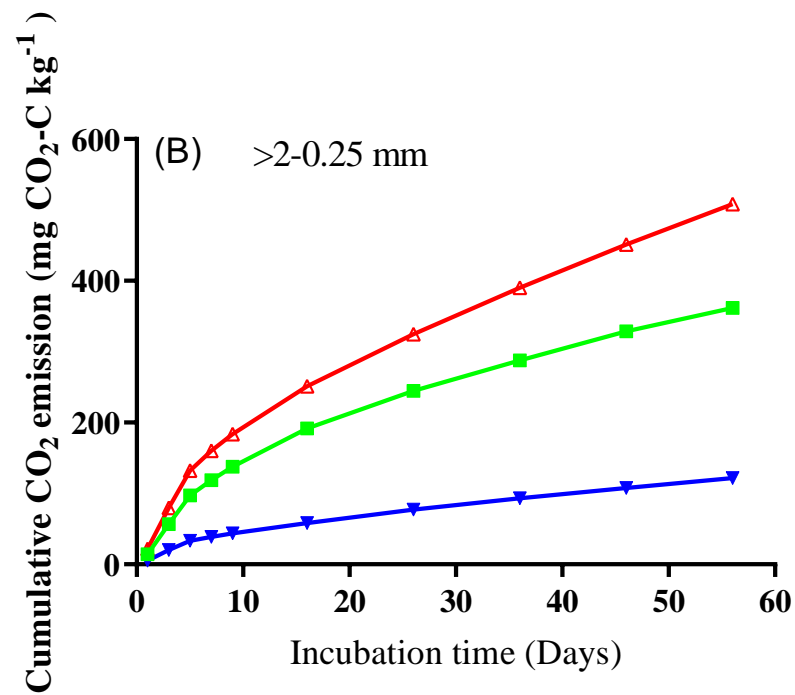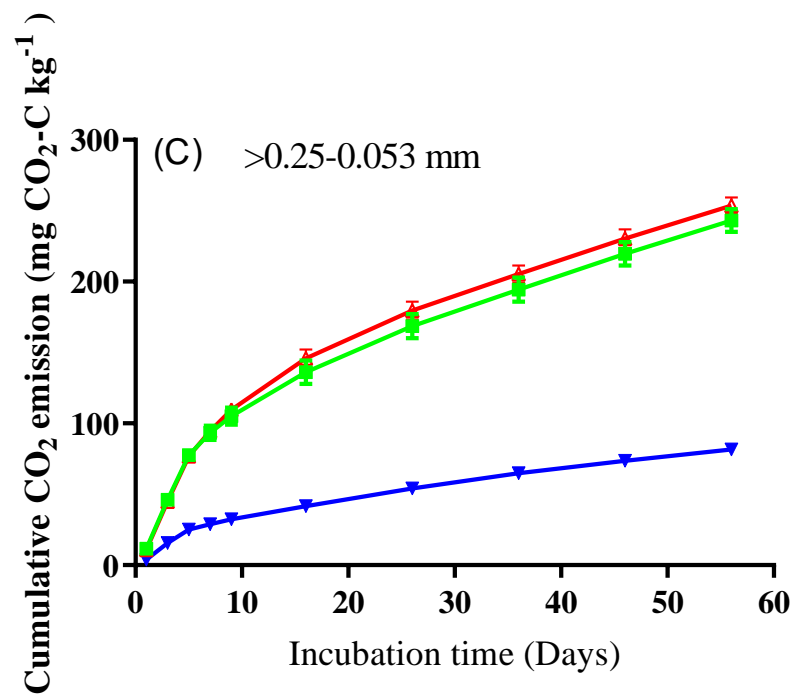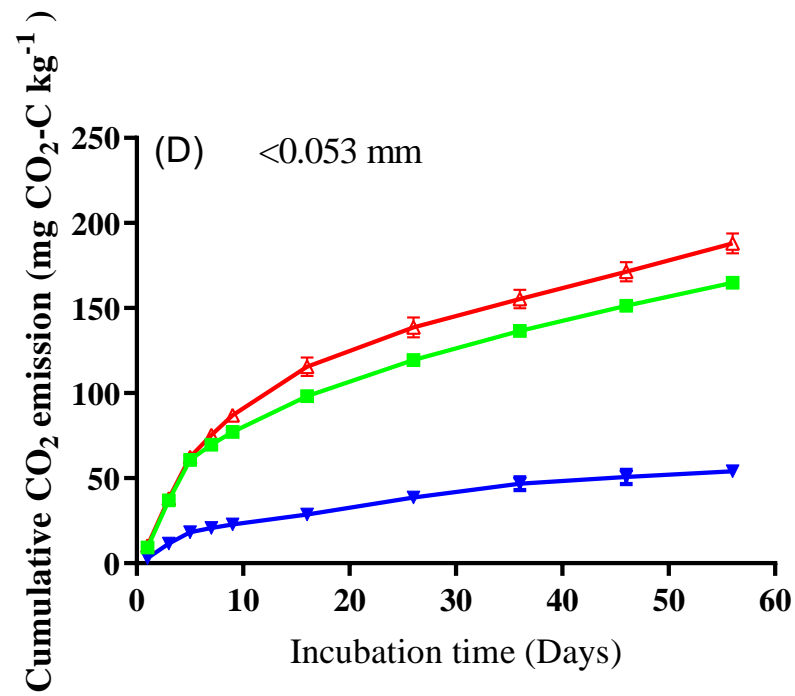

CK

GG

GC

Supplement: Supplemental Information 2 — CK, non-grazing natural grassland; GG, grazing grassland; GC, non-grazing natural grassland converted to artificial grassland. Error bars represent standard deviation (n = 4). [file peerj-09-11807-s002.pdf]

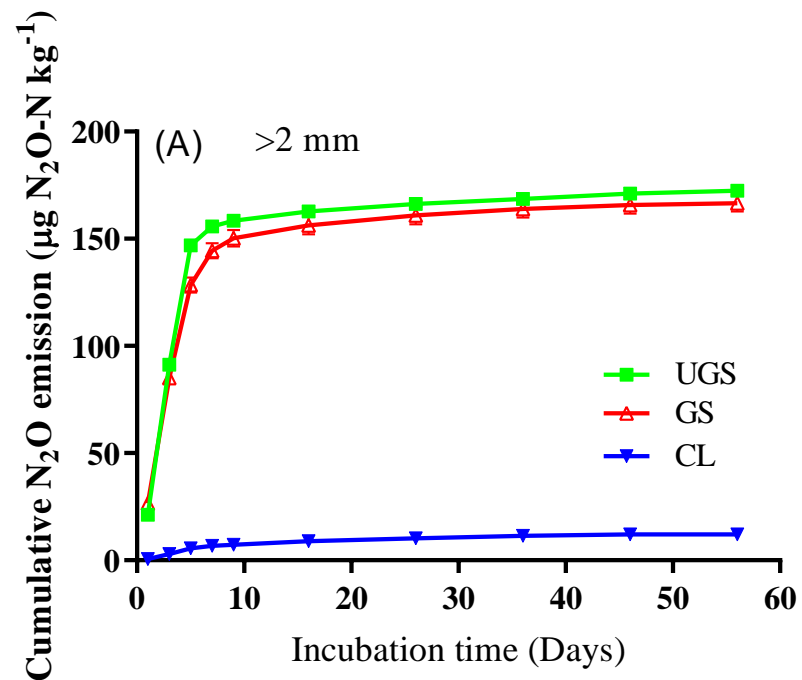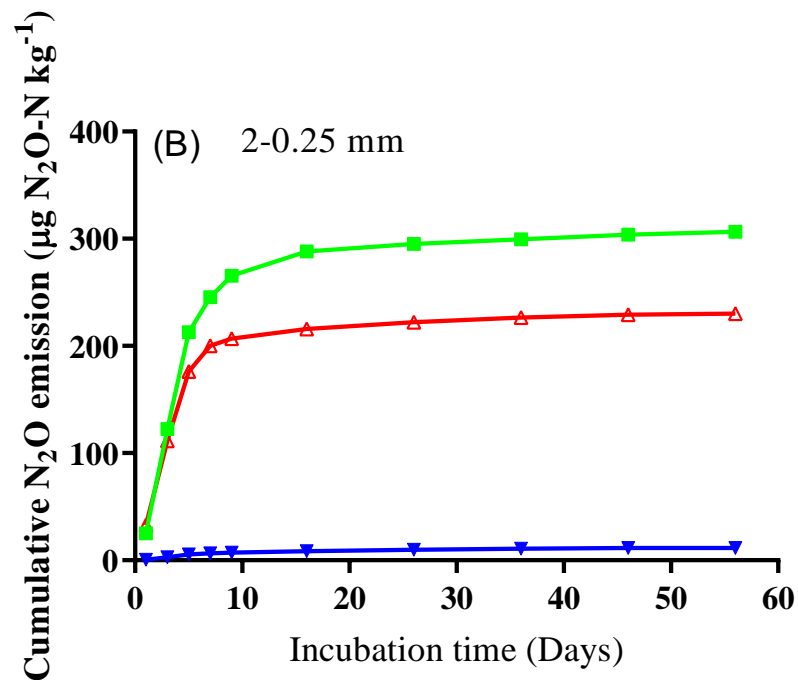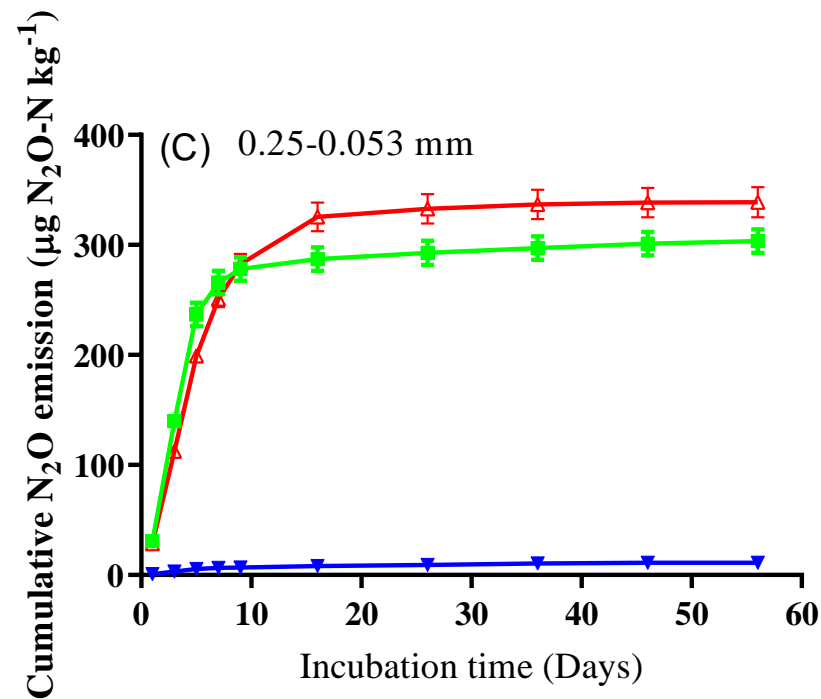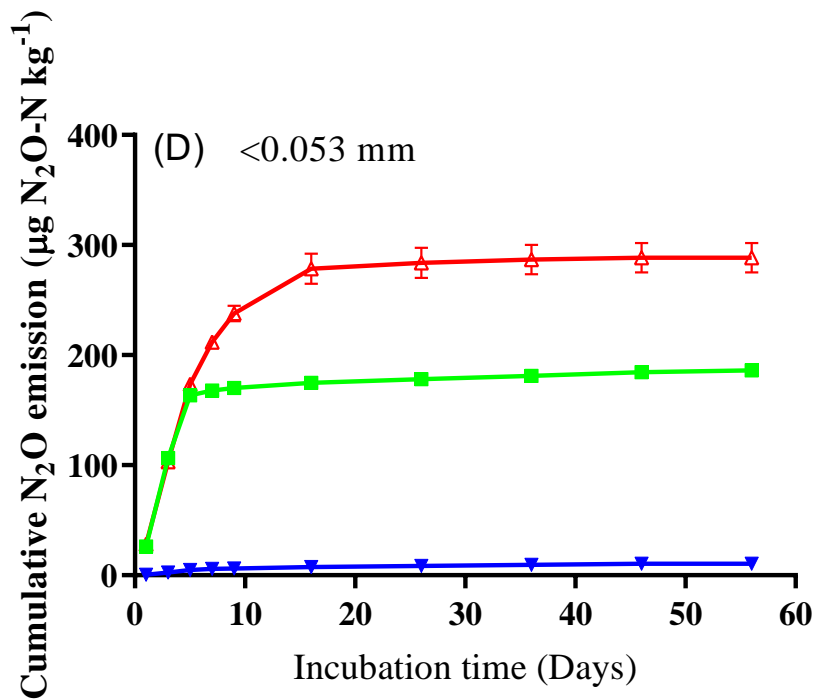

CK

GG

GC

Supplement: Supplemental Information 3 — CK, non-grazing natural grassland; GG, grazing grassland; GC, non-grazing natural grassland converted to artificial grassland. Error bars represent standard deviation (n = 4). [file peerj-09-11807-s003.pdf]
